# INCIDENCE OF RIFAMPICIN-RESISTANCE PRESUMPTIVE *M. TUBERCULOSIS* CASES AMONG OUTPATIENTS IN KEBBI STATE, NIGERIA

**DOI:** 10.21010/ajid.v15i1.6

**Published:** 2020-12-14

**Authors:** Mohammed Bashar Danlami, Basiru Aliyu, Grace Samuel

**Affiliations:** 1 Department of Microbiology, Federal University Birnin Kebbi, PMB 1157 Kebbi State, Nigeria; 2 Department of Microbiology, Faculty of Life Sciences, Kebbi State University of Science and Technology, Aliero, P.M.B. 1144. Birnin Kebbi, Kebbi State, Nigeria

**Keywords:** Rifampicin resistance, *Mycobacterium tuberculosis*, GeneXpert, Kebbi, Nigeria

## Abstract

**Background:**

The present study determined the incidence of rifampicin resistance *M. tuberculosis* among outpatients at the General Hospital Yauri, Kebbi State, Nigeria.

**Materials and Methods:**

The study is a cross-sectional study conducted from February 2018 to October 2019. Sociodemographic data were collected from hospital registration books. Rifampicin resistance *M. tuberculosis* was detected using GeneXpert Model GX-IV following manufacturers’ instruction. Descriptive statistics and logistic regression were computed using SPSS version 20. The results were presented as odds ratios with associated 95% confidence intervals, and P-value at 0.05.

**Result:**

Of the 837 samples, 65.8% (551/837) were males, and 34.2% (286/837) females, 11.4% (95/837) HIV-seropositive. *M. tuberculosis* was detected in 15.5% (130/837), of which 116/130 (89.23%) were males and 14/130 (10.77%) females. *M. tuberculosis*-HIV coinfection was detected in 9.47% (9/95) of HIV positive. Rifampicin resistance was observed in 1.3% (11/837), 7.7% (10/130) in *M. tuberculosis* patients and 1.05% (1/94) in HIV seropositive. In logistic regression, the odds ratio for having a rifampicin-resistant *M. tuberculosis* was 0.49 (0.15-1.54) for > 30 years; taking <30 years as the reference value, 1.02 (1.00-1.03) for male; taking female as the reference value, and 0.78 (0.09-6.15) for HIV positive, taking negative as the reference value.

**Conclusion:**

This study reported the current incidence rate of rifampicin-resistant *M. tuberculosis* at the General Hospital Yelwa Yauri, Kebbi State, Nigeria, among presumptive TB patients. Patients diagnosed with rifampicin-resistant *M. tuberculosis* were predominantly male adults. Thus, frequent screening is vital for surveillance and reduces the risk of transmission and spread of *M. tuberculosis* infections.

## Introduction

The spread of multidrug-resistant tuberculosis (MDR-TB) remains a public health problem in Nigeria (Audu *et al.*, 2017). The country is among the high-TB-endemic in the world. An estimated 4.3% of tuberculosis patients in the country are MDR, out of which 32% are newly diagnosed (WHO, 2016; Ukwamedua *et al.*, 2019). A recent national survey in Nigeria recorded a 2% increase in the prevalence of MDR-TB among pretreatment cases (Onyedum *et al.*, 2017). In Kebbi State, comprehensive information about the patterns of multidrug-resistant tuberculosis is lacking, and only a few studies have assessed the extent of multidrug-resistant tuberculosis among likely TB patients in other states of the federation ( Aminu and Tukur, 2017; Onyedum *et al.*, 2017).

Rifampicin and isoniazid are essential drugs in the treatment of tuberculosis. The two drugs are critical for short-term treatment with reduced toxicity because of long-term therapy ( WHO, 2016; Ukwamedua *et al.*, 2019). Studies have shown that MDR-TB to rifampicin is accompanied mainly by resistance only to isoniazid (WHO, 2016; Ukwamedua *et al.*, 2019).

In the last few decades, culture is the most common laboratory diagnostic technique for *M. tuberculosis* (Ma *et al.*, 2006; Derseh *et al.*, 2017.) The technique requires sophisticated and expensive biosafety laboratory facilities (Arega *et al.*, 2019). Of recent, Gene Xpert assay, an automated molecular assay detects mutations on the *rpoB* gene responsible for 90% cases of rifampicin-resistance in TB patients with less sophisticated facilities (Okonkwo *et al.*, 2017; Onyedum *et al.*, 2017). Thus, the treatment of *M. tuberculosis* without susceptibility testing increases the risk of transmission and spread of MDR-TB strains in a population ( Xiao-li *et al.*, 2014; Onyedum *et al.*, 2017; WHO, 2018).

With the projected incidence of 219 new TB cases per 100,000 people per year in the country (Onyedum *et al.*, 2017; Ukwamedua *et al.*, 2019), screening for rifampicin-resistance tuberculosis within the population is vital for the treatment of MDR-TB. This study will provide up-to-date insight into the incidence and extent of rifampicin-resistance among likely TB patients in one of the referral hospitals in Kebbi State of Nigeria.

## Materials and Methods

### Study design, area and period

The study was conducted at General Hospital Yelwa Yauri, an Emirate Region situated in the southern part of Kebbi State, Nigeria, between February 2018 to October 2019. In Yelwa Yauri, 54% of the populations live in rural areas, and half of the communities are illiterates. The hospital is a secondary level health facility located in the heart of the town.

### Study design and Data collection

Participants microbiological and clinical information were collected from the hospital records for all the presumptive patients. Patients’ records with incomplete data, e.g., age, gender, Xpert MTB/RIF results, and HIV status and unsuccessful results were excluded from the study. Failed results were classified as invalid, error and undetermined. The inclusion criteria for this study included participants willing to provide their samples freely and those who presented themselves as patients with a long-term duration of cough and chest pain and patients that are booked for AFB screening.

### Gene Xpert assay

Single sputum samples per patient were used for the diagnosis of *Mycobacterium tuberculosis* using Gene Xpert assay. Samples were processed using Model GX-IV of the Gene Xpert (Cepheid Sunnyvale, CA, USA) following the manufacturer’s instruction. Briefly, 0.5 ml sputum sample and Xpert sample reagent were added in a ratio of 1:2, vortex twice for 15 min at room temperature. Consequently, 2 ml of the reaction mixture was transferred to the Xpert test cartridge; the cartridge was then loaded into the Xpert machine for 90 mins. The results were interpreted from measured fluorescent signals automatically as MTB detected, not detected and if present- rifampicin resistant.

### Data processing and statistical analysis

The database was generated in Microsoft Excel (Microsoft Corp., Redmond, WA, USA). Descriptive analysis was adopted using the Statistical Package for the Social Sciences (SPSS®, version 21; SPSS Inc., Chicago, IL, USA). Frequencies and percentages were calculated, and odds ratio and nominal 95% confidence intervals (CI) were presented to explain the association between variables - age, gender, marital status, RIF resistance and HIV status independently as against MTB detection. A two-sided *p-value* < 0.05 was considered significant.

### Ethical issue

This study was approved by the ethical research and review committee of the Ministry of Health Birnin Kebbi, Kebbi State, Nigeria (KSUSTA1510204007), on 02/05/2019.

## Results

Of the 896 patients that submitted samples for TB diagnosis, 82.2% (837/896) were included in the study. Five samples were external quality assurance (EQA) samples, 21 samples were invalid, 19 were error, and the remaining 14 samples were undetermined. Out of these 837, 65.8 % (551/837) were males, and 34.2 % (286/837) were females. The study population median age was 35 years, 27.7% (232/837) were in the age range of 21–30 years, while 2.4% (20/837) were in the age range under ten years. Among total study participants, 11.4% (95/837) were HIV-seropositive, and the remaining 88.6% (742/837) were HIV-seronegative (Table 1).

**Table 1:** Sociodemographic distribution and clinical characteristics of tuberculosis patients in (N = 837)

**Variables**	**Total Patients**	**Percentage %**
**Age**		
Under 10	20	2.4
11-20	105	15.5
21-30	232	27.7
31-40	176	21.0
41-50	143	17.1
51-60	100	11.9
Above 60	61	7.3
**Gender**		
Male	551	65.8
Female	286	34.2
**Marital Status**		
Single	233	27.8
Married	604	72.2
**MTB Status**		
Detected	130	15.5
Not Detected	707	84.5
**RIF Resistant**		
Detected	11	1.3
Not Detected	826	98.7
**HIV Status**		
Positive	95	11.4
Negative	742	88.6

Among the enrolled presumptive TB patients, MTB was detected in 15.5% (130/837), of which 116/130 (89.23%) were males, and 14/130 (10.77%) were females. Of presumptive MTB patients, the age group 31–40 years with a median age of 35 years, found prone to MTB infection 40/130 (30.8%). MTB-HIV coinfection was detected in 9.47% (9/95) of HIV positive patients, table 2. Rifampicin-resistant was observed in 1.3% (11/837) in the presumptive TB patient, while the total RIF-MTB was 7.7% (10/130) in all MTB detected patients and 1.05% (1/94) in HIV seropositive patient (Table 2).

**Table 2:** Characterization of *M. tuberculosis* in presumptive TB patients based on age, gender, HIV status and marital status in Kebbi State, Nigeria (*N* = 837)

Variables	Detected	Not Detected	OR(95%CI)	*P_Value*
Age* MTB				
Under 10	3	17	00*	
11-20	6	99	0.35(.08-1.50)	
21-30	33	199	0.94(0.26-3.39)	
31-40	40	136	1.67(0.47-6.0)	
41-50	25	118	1.20(0.33-4.41)	
51-60	13	87	0.85(0.22-3.30)	
Above 60	10	51	1.11(0.28-4.51)	
Gender* MTB				
Male	116	435	0.19(0.11-0.34)	.000
Female	14	272	00*	
Marital Status* MTB				
Single	18	215	00*	
Married	112	492	0.37(0.22-0.62)	.000
RIF Resistant* MTB				
Detected	10	120	0.02(0.002-0.13)	
Not Detected	1	706	00*	
HIV Status* MTB				
Positive	9	121	1.86(0.91-3.8)	0.09
Negative	86	621	00*	

00

* = Reference category

MTB = *M. tuberculosis*

OR: Odd ratio

In a logistic regression model, taking <30 years as the reference value, the odds ratio for having a rifampicin-resistant MTB was 0.49 (95% CI, 0.15-1.54) for age groups **>** 30 years, 1.02 (95% CI, 1.00-1.03) for male taking female as the reference value. It is 1.75 (95% CI, 0.33-8.17) for married, single as a reference value, and 0.78 (95% CI, 0.09-6.15) for HIV positive individuals, taking negative as the reference value (Table 3).

**Table 3:** Characterization of rifampicin-resistant *M. tuberculosis* in presumptive TB patients based on age, gender, HIV status and Marital Status in Kebbi State, Nigeria (*N* = 837).

**Variables**	**Detected**	**Not Detected**	**OR(95%CI)**	***P_Value***
Age* RIF				
<30 years	4	353	00*	
> 30 years	7	473	0.49(0.15-1.54)	0.29
Gender* RIF				
Male	11	540	1.02(1.00-1.03)	0.02
Female	0	286	00*	
Marital Status* RIF				
Single	2	231	00*	
Married	9	395	1.75(0.33-8.17)	0.74
HIV Status* RIF				
Positive	1	94	0.78(0.09-6.15)	1.00
Negative	10	732	00*	

00

* = Reference category

## Discussion

The spread of rifampicin resistant *M. tuberculosis* is a threat to treatment and control of tuberculosis (Onyedum *et al.*, 2017; WHO, 2018). Thus, early detection is essential for the management and prevention of transmission of the disease. In the present study, we combined sociodemographic data and Xpert MTB/RIF assay and assessed the incidence of rifampicin resistant *Mycobacterium tuberculosis* among outpatients referred to the General Hospital Yauri, Kebbi State, Nigeria. This study is first to describe the scale of *M. tuberculosis* cases in the study population.

The overall incidence rate of new *M. tuberculosis* cases in Yauri Emirate in Kebbi State was 15.5%. The result was slightly higher than the studies reported in Zaria (12%) in the North-west region of the country a decade ago ( Ogboi *et al.*, 2010). Though, the incidence is significantly lower than reports from previous studies in a referral hospital in South-west Nigeria (37.7%) (Adejumo *et al.*, 2018) and among patients previously treated for pulmonary tuberculosis in North-west of Nigeria (29.2%). The incidence is equally lower than it has been reported in another study in Nasarawa State in the North-central (18.8 %) (Audu *et al.*, 2017). This result is considerably lower when compared with the results obtained using a line probe assay from a referral hospital in Kano State (54.5 %) (Aminu and Tukur, 2017). The low incidence rate reported in this study compared with other studies could be attributed to sampling techniques, size and methodology adopted.

Similarly, this study reported a 1.3% incidence rate of rifampicin resistance (11/ 837). The rate is significantly lower than the 7.2% reported in Kwara State, Nigeria, 6.9% recorded in Nnewi, Nigeria (Dim and Dim, 2013; Okonkwo *et al.*, 2017). Also, the incidence rate is relatively below the projected rate of 3.2–5.4% for the country ( WHO, 2016; WHO, 2018). Although the resistance in this study is very low due to the sample size. The low incidence rate recorded may be due to (i) this study considered only presumptive TB cases from outpatients using gene Xpert assay, (ii) the rifampicin resistance positive cases recorded were infected with a resistant *M. tuberculosis* strain.

This study also found a high number of *M. tuberculosis* cases and rifampicin resistance among males compared to females. All the 10 rifampicin-resistant *M. tuberculosis* were detected in presumptive TB patients. Analysis of statistical significance reveals that gender is associated with rifampicin-resistant tuberculosis with p-values of 0.02. The result was in line with a high rate of multidrug resistance TB reported in men in North and southern regions of Nigeria ( Iliyasu and Babashani, 2009; Derseh *et al.*, 2017) and many African countries (Cox *et al.*, 2010; Kirenga *et al.*, 2015). Also, the result was consistent with other studies that stated that the male gender is at risk of developing drug resistance due to behavioural factors such as poor health-seeking behaviour, not finishing an antibiotic course and cultural activities that expose male gender to infectious agents (Bello and Itiola, 2010).

The average age range with the highest rate of *M. tuberculosis* cases was 31-40 years, and rifampicin resistance was in > 30 years. This result correlates with other studies reported in Nigeria, where patients between 20-40 years had a higher prevalence of rifampicin resistance. However, the results disagree with studies that reported younger age had been associated with PTB and MDR-PTB (Onyedum *et al.*, 2017; Ukwamedua *et al.*, 2019). The distinction observed in this study was 48% of the total population in this study were adults between 20-40 years of age and 72.5% rural farmers (data not shown) associated with unpasteurized cow milk in local porridge as a staple food in the farm. The unpasteurized cow milk was the primary source of *M. tuberculosis* to the farmers (Araújo *et al.*, 2014.; Lorente-Leal *et al.*, 2019).

The association between MTB-HIV coinfection has long been established (Oshi *et al.*, 2014). Despite this, a low rate of MTB-HIV coinfection was reported in this study. However, the reduced rates of MTB-HIV coinfection recorded in this study may be because a significant number of the study participants had no contact with HIV because of increasing public enlightenment in the population ( Keating *et al.*, 2006; Gambo *et al.*, 2013).

## Conclusion

This study reported the current incidence rate of rifampicin-resistant *M. tuberculosis* at General Hospital Yelwa Yauri Kebbi State, Nigeria, among possible *M. tuberculosis*. The patients diagnosed with rifampicin-resistant *M. tuberculosis* were predominantly adult males. This result demonstrated that GeneXpert assay is a convenient tool for the early diagnosis of rifampicin-resistant *M. tuberculosis*. Accordingly, frequent screening and surveillance within the population are vital for the management and treatment of *M. tuberculosis* infections.

**List of Abbreviations:**
HIV: : Human immunodeficiency virus
MDR-TB: : Multidrug-resistant tuberculosis
MTB: : *M. tuberculosis*
RIF: : Rifampicin-resistant
TB: : tuberculosis
CI: : Confidence interval
OR: : Odd ratio
EQA: : External quality assurance
WHO: : World Health Organization
SPSS: : Statistical package for social sciences
